# High Prevalence of Problematic Health Literacy and Its Associated Sociodemographic Factors Among Community-Dwelling Individuals in Northern Thailand: A Cross-Sectional Study

**DOI:** 10.3390/ijerph23060752

**Published:** 2026-06-03

**Authors:** Uratcha Sadjapong, Nattapon Harnsamut, Patipat Vongruang, Sakesun Thongtip

**Affiliations:** 1 Department of Health Promotion, School of Public Health, University of Phayao, Phayao 56000, Thailand; uratcha.sa@up.ac.th; 2 Department of Software Engineering, School of Information and Communication Technology, University of Phayao, Phayao 56000, Thailand; nattapon.ha@up.ac.th; 3 Department of Environmental Health, School of Public Health, University of Phayao, Phayao 56000, Thailand; patipat.vo@up.ac.th

**Keywords:** health literacy, sociodemographic factors, rural Thailand, older adults, health disparities

## Abstract

**Highlights:**

**Public health relevance—How does this work relate to a public health issue?**

- This study identifies a high prevalence of problematic health literacy (HL) among community-dwelling adults in Chiang Muan District, Northern Thailand.
- The findings highlight limited HL as a critical and systemic public health challenge in rural communities.

**Public health significance—Why is this work of significance to public health?**

- Problematic HL was consistently observed across all key health dimensions, including health care, disease prevention, and health promotion.
- The results reveal widespread challenges in all health information-processing competencies: accessing, understanding, appraising, and applying health information.

**Public health implications—What are the key implications or messages for practitioners, policy-makers and/or researchers in public health?**

- The findings support the urgent development of targeted, context-specific HL interventions tailored for rural populations, especially those with lower educational attainment.
- Such interventions should be delivered through plain-language communication, primary care services, village health volunteers, and community-based health promotion activities.

**Abstract:**

Health literacy (HL) is an important factor associated with individuals’ capacity to manage their health effectively. This cross-sectional study assessed HL and its associated factors among 327 community-dwelling individuals in Northern Thailand. Data were collected through face-to-face interviews using the 47-item European Health Literacy Questionnaire (HLS-EU-Q47). The participants were predominantly female (59.9%), with a mean age of 59.0 ± 11.7 years. Overall, 60.2% of the participants exhibited problematic HL across all domains. In bivariate analyses, overall HL was significantly associated with sex, age, body mass index (BMI), education, marital status, and hypertension. Age was negatively correlated with overall HL (r = −0.250, *p* < 0.001), whereas BMI was positively correlated with overall HL (r = 0.130, *p* = 0.019). In the multivariable linear regression model, higher education (β = 4.251, *p* < 0.001), female sex (β = 2.310, *p* = 0.002), and alcohol consumption (β = 1.411, *p* = 0.047) were independently associated with higher HL scores. Conversely, marital status (β = −1.747, *p* = 0.033) was associated with lower HL scores. Problematic HL was highly prevalent in this population and was associated with sociodemographic and health-related factors. These findings highlight the need for targeted, context-specific health communication and education strategies to improve HL among vulnerable community-dwelling populations, particularly older adults and individuals with lower educational attainment.

## 1. Introduction

Health literacy (HL) has been widely recognized as an important social determinant of health outcomes and health equity [1,2]. The World Health Organization (WHO) defines HL as the ability of individuals to access, understand, appraise, and apply health information in ways that promote and maintain good health [1]. Beyond individual knowledge, HL reflects the interaction between personal competencies, health communication, and the complexity of health systems [1]. Adequate HL enables individuals to make informed decisions, engage in preventive behaviors, and navigate healthcare services effectively, whereas limited HL is associated with poorer health behaviors, reduced use of preventive services, and increased risk of adverse health outcomes [2,3].

Globally, limited HL remains a major public health challenge, particularly amid population aging and the growing burden of chronic diseases [4,5]. Individuals with higher HL are more likely to engage in self-care, adhere to treatment recommendations, and seek timely medical attention, which may contribute to better disease prevention and management [6,7,8]. However, HL is not distributed equally across populations [9]. Previous studies have shown that older adults, individuals with lower educational attainment, and socioeconomically disadvantaged groups are more likely to experience inadequate or problematic HL [10,11,12]. These inequalities may further reinforce disparities in healthcare access, service utilization, and health outcomes [13,14].

In Thailand, non-communicable diseases (NCDs), including cardiovascular diseases, cancer, diabetes, and chronic respiratory diseases, remain a major public health concern [15,16]. Although the country has made substantial progress in universal health coverage, inequities in health knowledge, access to reliable health information, and effective use of healthcare services persist, especially among rural and community-dwelling populations [17,18]. In such contexts, HL becomes particularly important because individuals are expected to interpret health information, make preventive decisions, and manage chronic disease risks within their everyday social and community environments [2,19].

Previous Thai studies have examined HL in different population groups; however, evidence remains limited regarding comprehensive HL assessed across multiple domains among rural community-dwelling adults in Northern Thailand [7,20,21]. In particular, less is known about how sociodemographic and health-related characteristics are associated with HL in rural districts where aging, lower educational attainment, and unequal access to health information may coexist. This gap limits the ability of public health practitioners to design targeted and context-specific HL interventions for vulnerable communities.

Therefore, this study aimed to assess the prevalence of comprehensive HL and examine factors associated with HL among community-dwelling individuals in Phayao Province, Northern Thailand. By addressing this context-specific knowledge gap, the findings may inform the development of targeted health communication strategies and community-based interventions to improve HL and promote more equitable health outcomes.

## 2. Materials and Methods

### 2.1. Study Design and Sampling

A cross-sectional study was conducted between September and October 2025 to assess HL among the community-dwelling adults in Chiang Muan District, Phayao Province, Northern Thailand. A multistage random sampling technique was employed to improve the representativeness of the sample. The sampling frame was based on the official household and resident lists obtained from local administrative records and community health databases in the study area. In the first stage, sub-districts were randomly selected. In the second stage, villages within each selected sub-district were randomly chosen. In the final stage, eligible individuals aged 18 years and older were randomly recruited from the household or resident lists of each selected village. If selected individuals were unavailable or declined to participate, replacement participants were selected using the same random sampling procedure from the remaining eligible individuals in the sampling frame. Information on the exact number of individuals approached and refusals was unavailable; therefore, the response rate could not be calculated. A total of 327 participants completed the survey.

The sample size for this study was determined using the finite population proportion formula—this calculation was based on a desired margin of error of 5%, a significance level (α) of 0.05, and a corresponding Z-score of 1.960. The estimation was further informed by a previously reported HL prevalence of 24.3% from a study conducted in Thailand [20]. Based on a total target population of 7138 individuals, the minimum required sample size was 327 participants.

### 2.2. Data Collection

Data were collected through face-to-face interviews conducted by trained interviewers who had received standardized training on the research instrument prior to data collection. Eligible participants were community-dwelling adults aged 18 years and older who had lived in the study area for at least six months and were able to communicate in Thai or the local language. Individuals were excluded if they had severe communication impairment, cognitive impairment, or diagnosed neurological disorders that could prevent them from understanding or responding to the questionnaire. These conditions were assessed through initial screening by trained interviewers, consultation with village health volunteers or, when available, community health records, and confirmation of the participant’s ability to understand the study information and provide informed consent.

### 2.3. Instruments

The questionnaire consisted of three sections: (1) demographic characteristics, including sex, age, education level, and marital status; (2) health status, including BMI, alcohol consumption, smoking status, and the occurrence of diabetes mellitus (DM) and hypertension; and (3) HL, which was assessed using the Thai version of the HLS-EU-Q47. No additional translation was performed in the present study. The original HLS-EU-Q47 has been validated in several Asian countries and has been reported as a satisfactory comprehensive instrument for assessing HL in Asian populations [22,23]. Additionally, the questionnaire demonstrated excellent internal consistency, as indicated by a Cronbach’s alpha coefficient of 0.962. Each item is rated on a four-point Likert scale, with response options ranging from 1 (very difficult) to 4 (very easy). The HLS-EU-Q47 is based on a comprehensive conceptual model of HL and evaluates four core competencies related to health information processing: accessing, understanding, appraising, and applying. These competencies are assessed across three domains: health care, disease prevention, and health promotion.

The overall HL index is derived from seven sub-indices. Four sub-indices correspond to the core competencies—access (13 items), understanding (11 items), appraisal (12 items), and applying (11 items)—while three sub-indices correspond to the domains of health care (16 items), disease prevention (15 items), and health promotion (16 items). To ensure comparability, the HL indices are standardized to a unified scale ranging from 0 to 50 using the following formula:

HL Index = (mean − 1) × (50/3)

The resulting index scores are categorized into four levels of HL: 0–25 indicates inadequate HL, >25–33 indicates problematic HL, >33–42 indicates sufficient HL, and >42–50 indicates excellent HL.

### 2.4. Data Analysis

Descriptive statistics were used to summarize participant characteristics and HL levels, including means, standard deviations (SD), frequencies, and percentages. Differences in HL across categorical variables, including sex, age, body mass index (BMI), education level, marital status, alcohol consumption, smoking status, DM, and hypertension, were examined using independent-samples *t*-tests and one-way analysis of variance (ANOVA), as appropriate. Pearson’s correlation coefficient was used to assess associations between continuous variables. Multiple linear regression analysis was performed to identify factors associated with overall HL. Categorical variables were coded as follows: sex was coded as men = 0 and women = 1; marital status was coded as unmarried (single, widowed, divorced, or separated) = 0 and married = 1; education level was coded as ≤primary school = 0 and >primary school = 1; alcohol consumption was coded as no = 0 and yes = 1; smoking status was coded as no = 0 and yes = 1; DM was coded as no = 0 and yes = 1; and hypertension was coded as no = 0 and yes = 1. Age and BMI were entered as continuous variables. All relevant sociodemographic and health-related variables were included in the model. Assumptions of linear regression, including normality, linearity, homoscedasticity, and multicollinearity, were assessed prior to analysis. Multicollinearity was assessed using variance inflation factors (VIFs), with VIF values below 5 considered acceptable. The normality of residuals was examined using visual inspection of Q–Q plots, while homoscedasticity and linearity were assessed using residual plots. Model fit was evaluated using R^2^ and adjusted R^2^. Statistical significance was set at *p* < 0.05. All statistical analyses were performed using SPSS version 26.0 (IBM Corp., Armonk, NY, USA).

## 3. Results

### 3.1. Sociodemographic and Health-Related Characteristics of Participants Among Community-Dwelling Populations

The analysis of the 327 participants indicated that the majority were female (59.9%). The mean age was 59.00 years (SD = 11.69), with more than half of the participants aged ≤60 years (56.0%). The mean BMI was 23.96 (SD = 4.06), and most participants were classified as having a normal weight (58.4%). Regarding educational attainment, the majority had completed primary school or had a lower level of education (59.9%). Most participants were married (82.6%). Regarding health-related behaviors, over half of the participants reported not consuming alcohol (54.7%). Additionally, the majority did not have diabetes mellitus (88.1%) or hypertension (67.9%) (Table 1).

### 3.2. Health Literacy Levels Across Domains Among Community-Dwelling Populations

Overall, problematic HL was the most common level among participants, accounting for 60.2% of the sample. A similar pattern was observed across all three HL domains—health care, disease prevention, and health promotion, as well as across the four competency dimensions of accessing, understanding, appraising, and applying health information. These findings indicate that limited HL was not confined to a specific domain but was consistently observed across multiple aspects of health information processing (Table 2).

### 3.3. Associations Between Sociodemographic and Health-Related Characteristics and Health Literacy Among Community-Dwelling Populations

In the bivariate analyses, overall HL scores differed significantly by sex, age group, BMI category, education level, marital status, and hypertension status. Participants with higher educational attainment had notably higher HL scores than those with primary education or lower. Younger participants and those without hypertension also showed higher mean HL scores. In contrast, alcohol consumption, smoking status, and diabetes mellitus were not significantly associated with overall HL in the bivariate analyses (Table 3).

### 3.4. Correlations Between Age, BMI, and Health Literacy Domains Among Community-Dwelling Populations

Age was negatively correlated with overall HL and all HL domains, indicating that older participants tended to report lower HL scores. BMI showed weak positive correlations with overall HL and all HL domains. (Table 4).

### 3.5. Factors Associated with Overall Health Literacy Among Community-Dwelling Populations

In the multivariable linear regression model, education level remained the strongest factor associated with overall HL. Participants with higher education had significantly higher HL scores after adjustment for sociodemographic and health-related variables. Alcohol consumption was also positively associated with HL, although this finding should be interpreted with caution. Sex and marital status were independently associated with HL scores, whereas age, BMI, smoking status, diabetes mellitus, and hypertension were not statistically significant in the adjusted model (Table 5).

## 4. Discussion

This study provides important evidence regarding HL among community-dwelling individuals in Northern Thailand, revealing that most participants (60.2%) exhibited problematic HL across all domains. This finding is consistent with international literature using the HLS-EU-Q47, which has documented substantial proportions of limited HL in developed and developing countries [23,24]. For instance, the European HL Survey reported that nearly half of adults in several European countries had inadequate or problematic HL, underscoring that limited HL is a widespread global public health concern [3,24]. Similarly, studies conducted in Asian contexts, including Thailand and neighboring countries, have reported moderate-to-low HL levels, particularly among rural and aging populations [20,25]. Furthermore, enhancing HL has been shown to reduce the risk of developing non-communicable chronic diseases, highlighting the urgency of addressing these deficits [26].

The predominance of problematic HL across all domains in this study suggests that limitations in HL are not domain-specific but rather reflect systemic challenges in accessing, understanding, appraising, and applying health information. This aligns with conceptual frameworks proposed by the WHO, which emphasize that HL is shaped not only by individual competencies but also by the complexity of health systems and communication environments [1,27]. Although this study did not directly measure structural or digital factors, the consistently low HL observed across domains suggests that future research should examine contextual factors, such as access to health information, digital access, and health-service communication [28,29].

Age was identified as an important determinant of HL, with older individuals demonstrating significantly lower HL levels. This finding is consistent with previous research indicating that HL tends to decline with age [30,31]. The observed pattern may be attributable to age-related cognitive changes, diminished information-processing capacity, and reduced engagement with digital health resources [20,32,33]. However, the relationship between age and HL remains complex and context-dependent and may be influenced by unmeasured confounding factors such as education and socioeconomic status [1,11].

Educational attainment emerged as the strongest predictor of HL in this study. Multiple linear regression analysis demonstrated that higher education levels were significantly associated with higher overall HL. This finding is consistent with global evidence indicating that education plays a critical role in developing the cognitive and literacy skills required to access, interpret, and apply health information effectively [1,21]. Individuals with higher educational attainment are more likely to possess essential competencies such as reading comprehension, critical thinking, and numeracy, which facilitate the processing of complex health information [4]. In contrast, the relatively low educational attainment observed in this study population may contribute to the high prevalence of limited HL. These findings highlight the importance of developing accessible and culturally appropriate health communication strategies tailored to populations with varying levels of education [23,34].

Differences between sexes were also observed, with women demonstrating higher HL levels than men. Multiple linear regression analysis confirmed that female sex was significantly associated with overall HL. This aligns with previous studies indicating that women are more likely to engage in health-seeking behaviors, utilize healthcare services, and actively search for health information [21,33,34]. Although prior research on sex and HL has occasionally yielded mixed results, numerous studies suggest that women frequently score higher in HL because they traditionally serve as the primary healthcare navigators and caregivers for their families, leading to greater interaction with the healthcare system [35,36,37].

Marital status was significantly associated with HL, with single individuals demonstrating higher HL compared with those who were married in the bivariate analysis. Multiple linear regression analysis confirmed that marital status was independently associated with overall HL in the adjusted model. Based on the coding used in the regression model, married participants had lower HL scores than unmarried participants after adjustment for other sociodemographic and health-related factors. One possible explanation is that unmarried individuals may develop stronger independent HL due to the need to manage their own health [37,38]. However, existing evidence on the relationship between marital status and HL remains inconsistent, with some studies reporting no significant association [39]. The higher levels of HL among unmarried individuals in this cohort may also reflect unmeasured contextual or household-related differences; therefore, this finding should be interpreted cautiously rather than as a direct effect of marital status itself [1,10].

Although BMI was significantly associated with HL, the relationship was relatively weak. Interestingly, individuals with higher BMI demonstrated slightly higher HL levels, which contrasts with several previous studies reporting lower HL among individuals with obesity [40,41]. One possible explanation for this discrepancy is that individuals with higher BMI may have more frequent interactions with healthcare providers, thereby increasing their exposure to health information and, subsequently, enhancing their HL [41,42].

Alcohol consumption was positively associated with overall HL in the multivariable linear regression model. However, this finding should be interpreted with caution and should not be taken to imply that alcohol consumption improves HL. Although alcohol use is generally considered a health-risk behavior, its positive association with HL in this study may reflect residual confounding by unmeasured social and contextual factors, such as socioeconomic status, occupational status, income, social participation, or access to health information [43,44,45]. In rural Thai contexts, alcohol consumption may also be more common among socially active or income-earning groups, which could partly explain the observed association. Therefore, alcohol consumption in this study should be interpreted as an associated factor rather than a causal determinant of higher HL. Future studies should assess drinking frequency, quantity, drinking patterns, and socioeconomic context to clarify this relationship.

Furthermore, hypertension was associated with lower HL in the bivariate analysis; however, this association was not statistically significant after adjustment in the multivariable model. This finding is generally consistent with existing evidence suggesting that limited HL may be linked to poorer chronic disease management and adverse health outcomes [46,47]. Individuals with low HL often face difficulties in understanding medical instructions, adhering to treatment regimens, and engaging in effective self-care behaviors [6,47]. Finally, the high correlations observed across HL domains should be interpreted descriptively, as the overall HL score was derived from these component domains. These findings remain consistent with integrated models of HL, in which the competencies of accessing, understanding, appraising, and applying health information are conceptually interconnected [4,24].

This study has several limitations that should be considered when interpreting the findings. First, the cross-sectional design limits the ability to establish causal relationships or determine the temporal direction between HL and its associated factors. Second, some variables were self-reported and may therefore be affected by recall bias or social desirability bias. Third, although multistage random sampling was employed, the study was conducted in only one district of Northern Thailand; thus, the findings may not be generalizable to the broader Thai population. Fourth, potentially relevant factors, such as digital access, health service utilization, and social support, were not measured. Finally, the positive association between alcohol consumption and HL should be interpreted with caution because drinking frequency, quantity, and patterns were not assessed.

## 5. Conclusions

In conclusion, this study identified a high prevalence of problematic HL among community-dwelling individuals in Northern Thailand, with limited HL observed consistently across all core domains. Overall, HL was associated with several sociodemographic and health-related characteristics, particularly educational attainment, sex, marital status, and alcohol consumption in the adjusted model. However, these associations should be interpreted with caution, given the cross-sectional design and the possibility of residual confounding.

These findings suggest that HL improvement should be integrated into community-based public health strategies and routine primary care services. Public health policies should prioritize adults with lower educational attainment and promote plain-language communication, culturally appropriate health education materials, and locally accessible channels such as primary care units, village health volunteers, and community health promotion activities. At the health-system level, integrating HL-sensitive communication and HL screening into primary care and NCD services may help reduce communication barriers and promote more equitable access to health information among rural populations.

## Figures and Tables

**Table 1:** Sociodemographic and health-related characteristics of participants (*n* = 327).

Variables	Frequency	Percentage
Sex		
Women	196	59.9
Men	131	40.1
Age (mean = 59.00, SD = 11.689)		
>60 years	144	44.0
≤60 years	183	56.0
BMI (mean = 23.96, SD = 4.058)		
Underweight (<18.5)	17	5.2
Healthy weight (18.5–<25)	191	58.4
Overweight (25–<30.0)	98	30.0
Obesity (≥30)	21	6.4
Education level		
≤Primary school	196	59.9
>Primary school	131	40.1
Marital status		
Single	57	17.4
Married	270	82.6
Alcohol consumption		
No	179	54.7
Yes	148	45.3
Smoking status		
No	284	86.9
Yes	43	13.1
DM		
No	288	88.1
Yes	39	11.9
Hypertension		
No	222	67.9
Yes	105	32.1

**Table 2:** HL levels across domains among study participants (*n* = 327).

Variables	Frequency	Percentage
Overall (mean = 32.87, SD = 6.45)		
Inadequate HL	52	15.9
Problematic HL	197	60.2
Sufficient HL	50	15.3
Excellent HL	28	8.6
Health care (mean = 32.85, SD = 6.95)		
Inadequate HL	49	15.0
Problematic HL	199	60.9
Sufficient HL	49	15.0
Excellent HL	30	9.2
Disease prevention (mean = 32.90, SD = 6.90)		
Inadequate HL	47	14.4
Problematic HL	207	63.3
Sufficient HL	43	13.1
Excellent HL	30	9.2
Health promotion (mean = 32.91, SD = 6.61)		
Inadequate HL	41	12.5
Problematic HL	218	66.7
Sufficient HL	36	11.0
Excellent HL	32	9.8
Access (mean = 32.84, SD = 6.54)		
Inadequate HL	53	16.2
Problematic HL	195	59.6
Sufficient HL	50	15.3
Excellent HL	29	8.9
Understand (mean = 33.08, SD = 6.46)		
Inadequate HL	50	15.3
Problematic HL	196	59.9
Sufficient HL	55	16.8
Excellent HL	26	8.0
Appraise (mean = 32.46, SD = 6.74)		
Inadequate HL	50	15.3
Problematic HL	204	62.4
Sufficient HL	45	13.8
Excellent HL	28	8.6
Applying (mean = 33.14, SD = 6.51)		
Inadequate HL	45	13.8
Problematic HL	208	63.6
Sufficient HL	44	13.5
Excellent HL	30	9.2

**Table 3:** Sociodemographic and health-related characteristics and their association with overall HL (*n* = 327).

Variables	Mean	SD.	Overall HL, *p* Value
Sex			0.011 ^a^*
Women	33.59	6.79	
Men	31.80	5.76	
Age (mean = 59.00, SD = 11.689)			<0.001 ^a^***
>60 years	31.29	6.56	
≤60 years	34.11	6.09	
BMI (mean = 23.96, SD = 4.058)			0.035 ^b^*
Underweight (<18.5)	28.55	6.25	
Healthy weight (18.5–<25)	32.91	6.66	
Overweight (25–<30.0)	33.43	5.73	
Obesity (≥30)	33.38	6.90	
Education level			<0.001 ^a^***
≤Primary school	30.93	5.17	
>Primary school	35.78	7.07	
Marital status			0.001 ^a^**
Single	35.32	7.83	
Married	32.51	6.00	
Alcohol consumption			0.192 ^a^
No	32.45	6.41	
Yes	33.38	6.48	
Smoking status			0.239 ^a^
No	33.06	6.29	
Yes	31.64	7.39	
DM			0.676 ^a^
No	32.93	6.45	
Yes	32.46	6.51	
Hypertension			0.017 ^a^*
No	33.45	6.47	
Yes	31.64	6.25	

^a^ Independent *t*-Test, ^b^ One-way ANOVA, * *p* value < 0.05, ** *p* value < 0.01, *** *p* value < 0.001.

**Table 4:** Correlations between Age, BMI, and health literacy domains (*n* = 327).

Variables	Overall HL *r*, *p* Value *	Health Care *r*, *p* Value *	Disease Prevention *r*, *p* Value *	Health Promotion *r*, *p* Value *	Access *r*, *p* Value *	Understanding *r*, *p* Value *	Appraising *r*, *p* Value *	Applying *r*, *p* Value *
Age	−0.250, <0.001	−0.264, <0.001	−0.227, <0.001	−0.217, <0.001	−0.228, <0.001	−0.233, <0.001	−0.290, <0.001	−0.259, <0.001
BMI	0.130, 0.019	0.115, 0.038	0.142, 0.010	0.113, 0.041	0.110, 0.047	0.124, 0.025	0.150, 0.007	0.128, 0.021

* Pearson’s correlation coefficient (*r*) and corresponding *p*-values are reported.

**Table 5:** Multiple linear regression analysis for sociodemographic and health-related characteristics associated with overall HL (*n* = 327).

Variables	Beta	SE	95% CI		*p* Value
			Lower Bound	Upper Bound	
Age	−0.024	0.033	−0.088	0.040	0.469
Sex	2.310	0.752	0.830	3.790	0.002 **
Marital status	−1.747	0.815	−3.350	−0.144	0.033 *
Education level	4.251	0.737	2.800	5.702	<0.001 ***
BMI	0.097	0.086	−0.071	0.266	0.256
Alcohol consumption	1.411	0.706	0.021	2.801	0.047 *
Smoking status	0.206	1.103	−1.965	2.377	0.852
DM	0.770	1.059	−1.314	2.854	0.468
Hypertension	−1.393	0.751	−2.870	0.084	0.064

* *p* value < 0.05, ** *p* value < 0.01, *** *p* value < 0.001.

## Data Availability

The data supporting the findings of this study are available from the corresponding author upon reasonable request.

## Abbreviations

The following abbreviations are used in this manuscript:

HL	Health literacy
HLS-EU-Q47	The 47-item European Health Literacy Questionnaire
WHO	World Health Organization
DM	Diabetes mellitus
SD	Standard deviations
BMI	Body mass index
One-way ANOVA	One-way analysis of variance
CI	Confidence interval
